# Supplementary figures and images for: Polydom/SVEP1 binds to Tie1 and promotes migration of lymphatic endothelial cells
Source: J Cell Biol. 2023 Jun 20;222(9):e202208047. doi: 10.1083/jcb.202208047 (PMC10281526; doi:10.1083/jcb.202208047)

Figure 3C

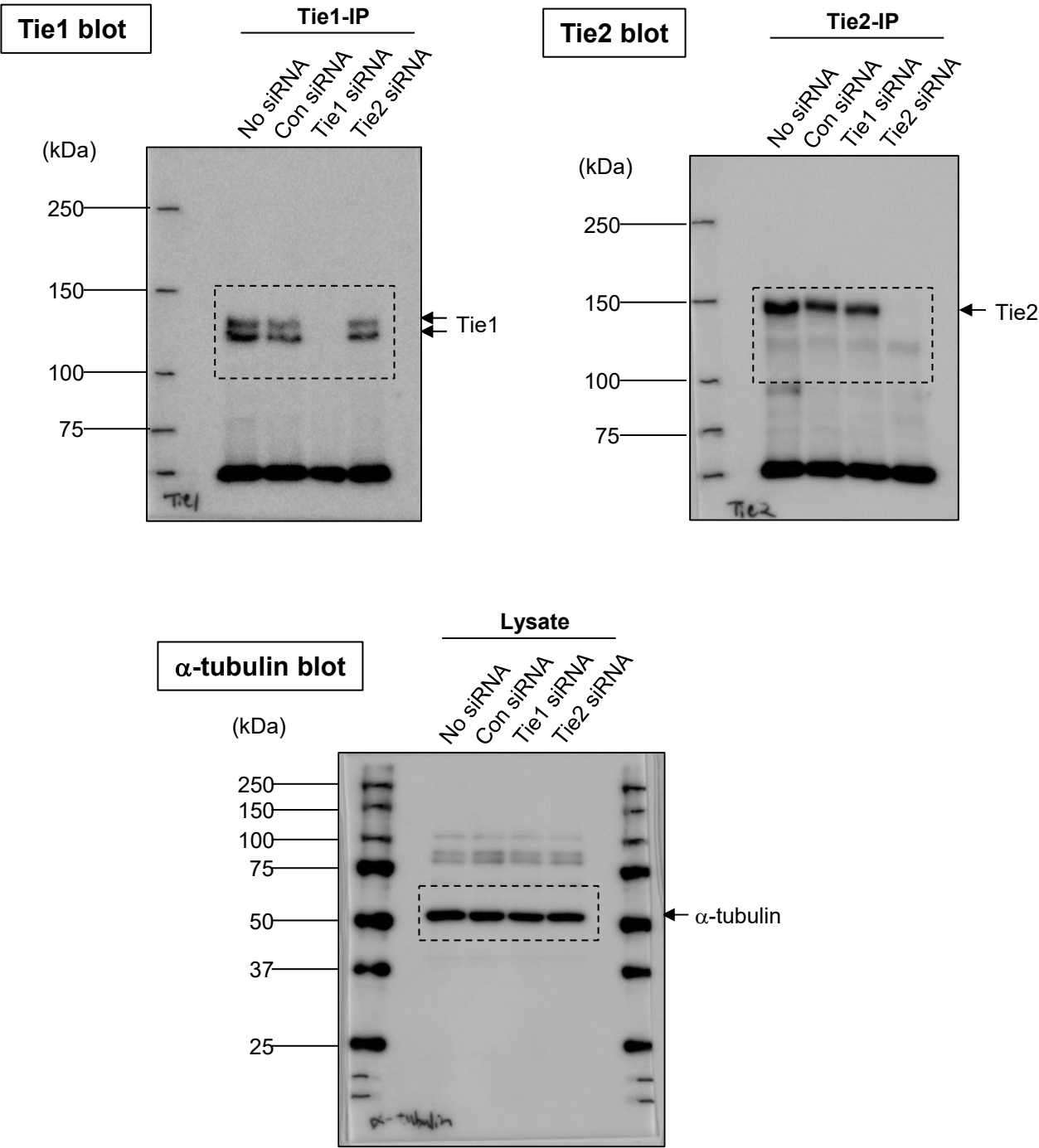

Figure 3F

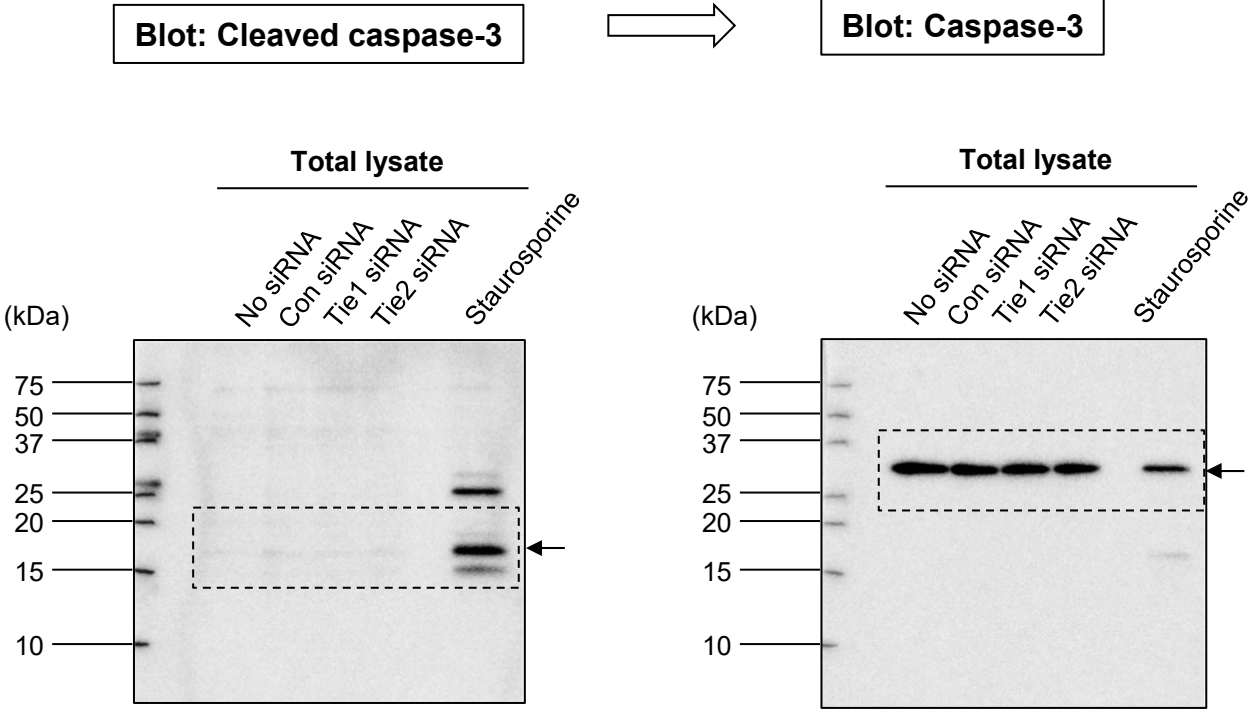

Supplement: SourceData F3 — is the source file for Fig. 3. [file JCB_202208047_SourceDataF3.pdf]

Figure 4B

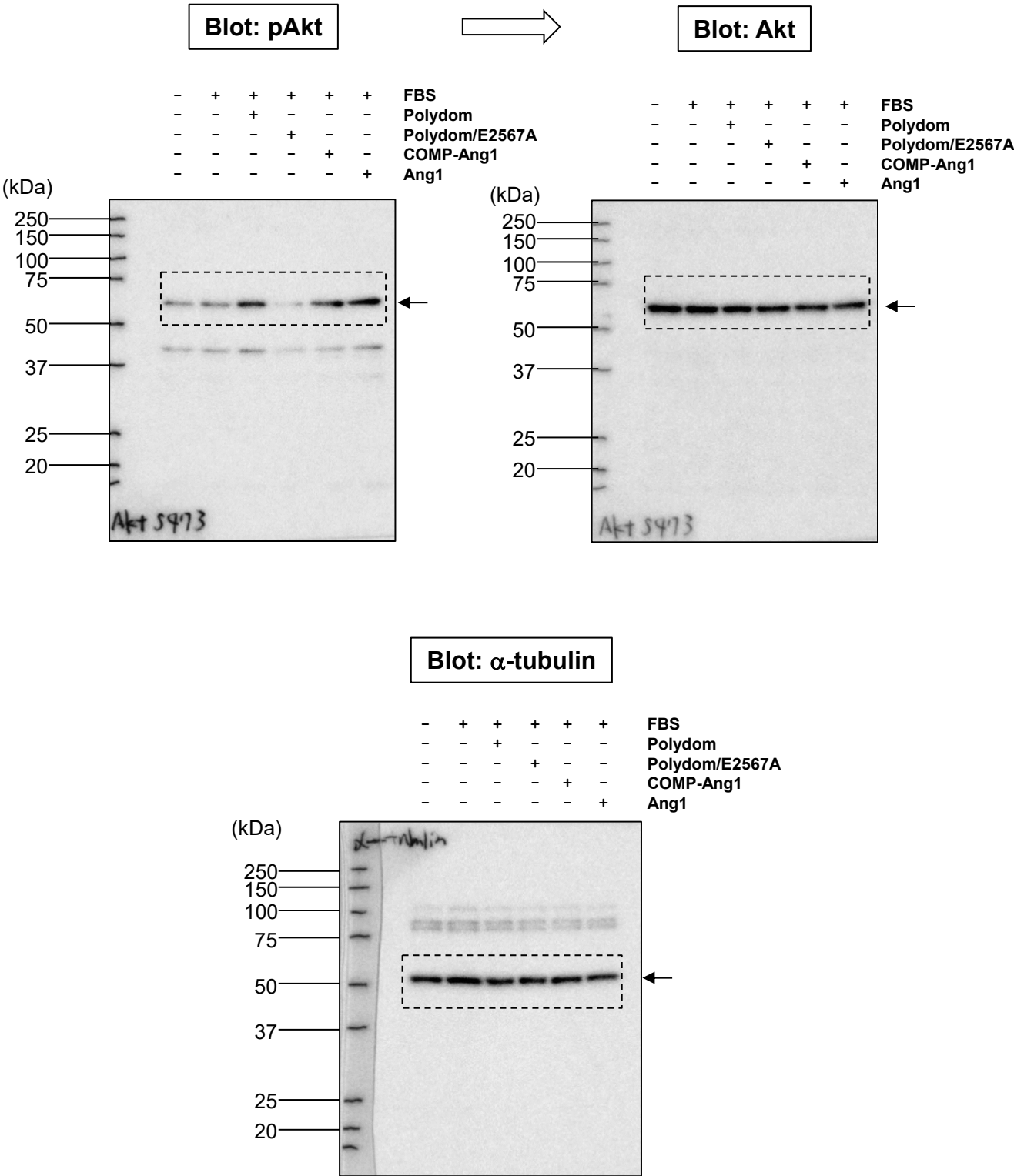

Supplement: SourceData F4 — is the source file for Fig. 4. [file JCB_202208047_SourceDataF4.pdf]

# Figure S3A Left

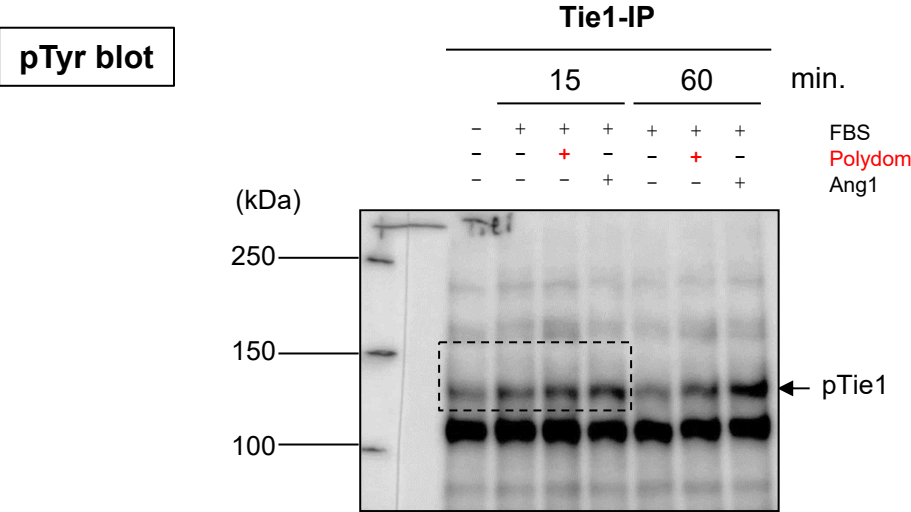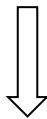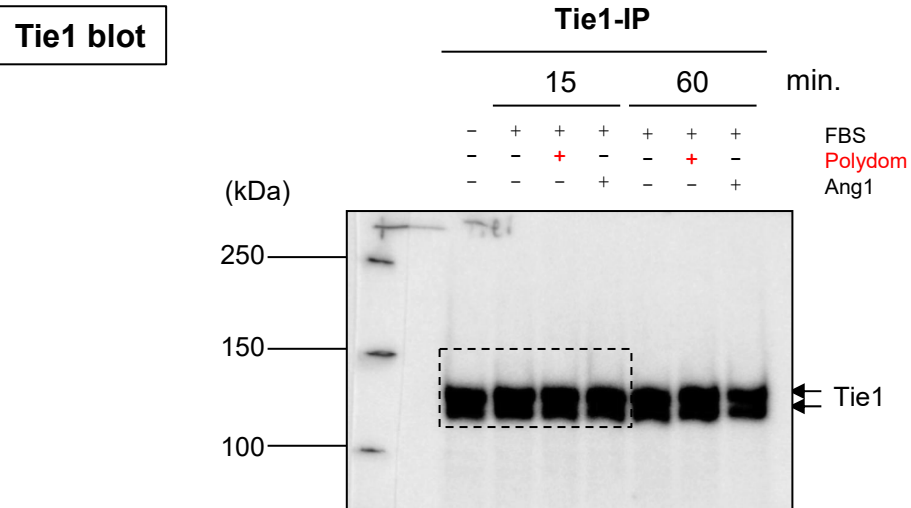

Figure S3A Right

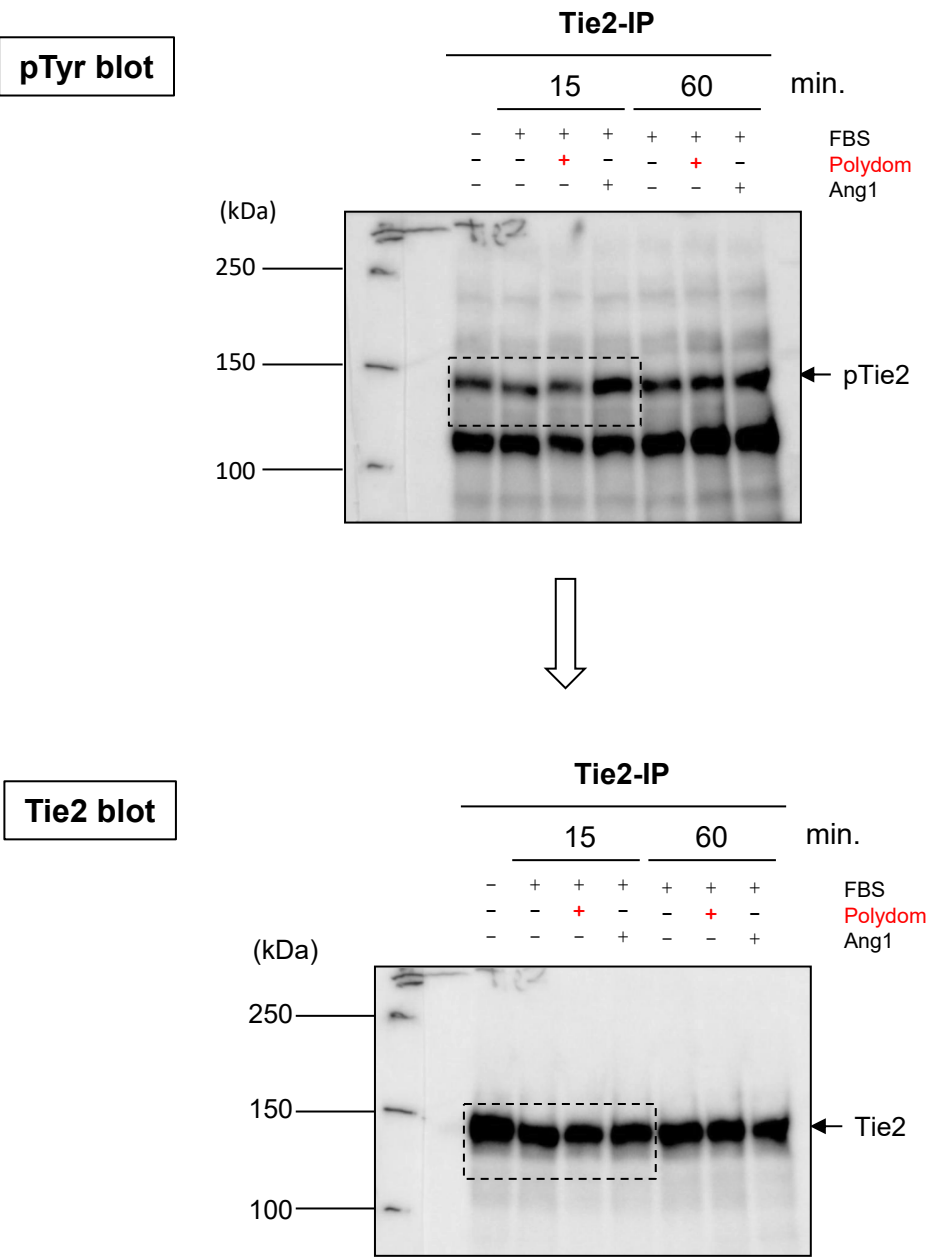

Figure S3B Upper

pTyr blot

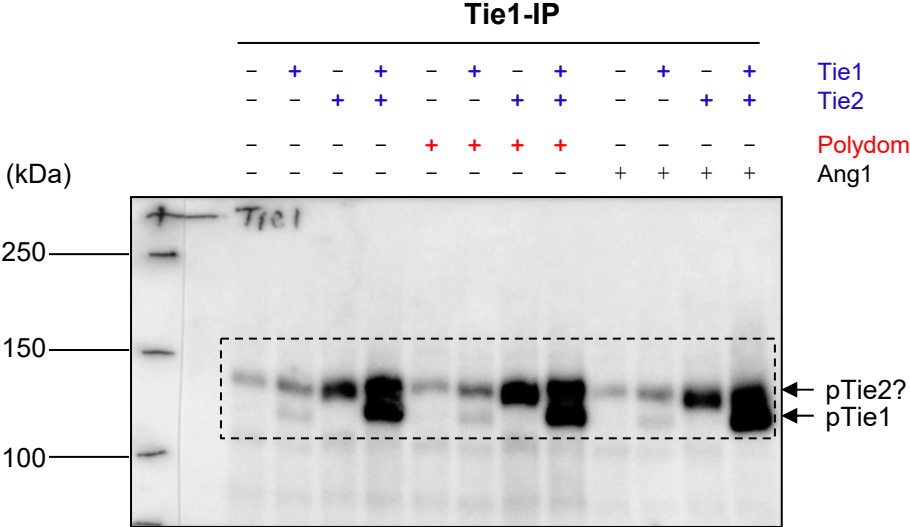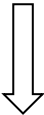

Tie1 blot

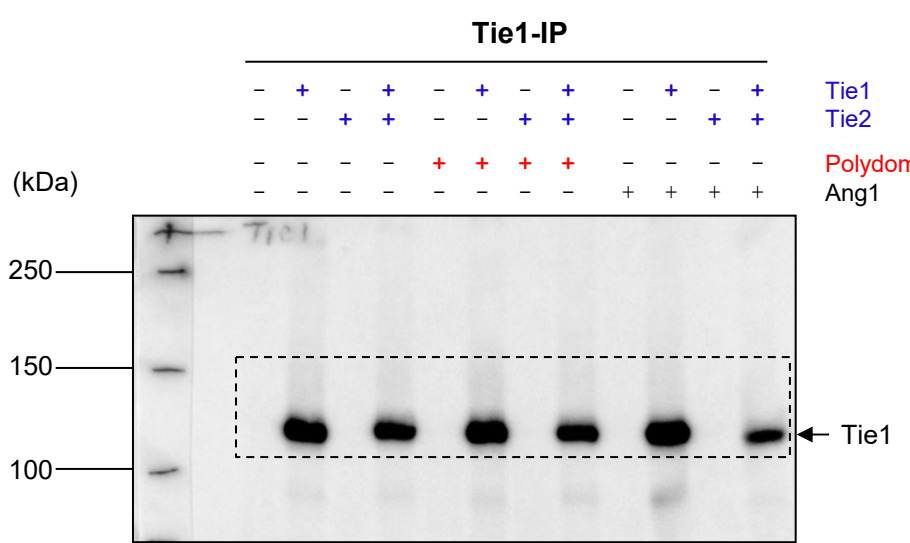

Figure S3B Lower

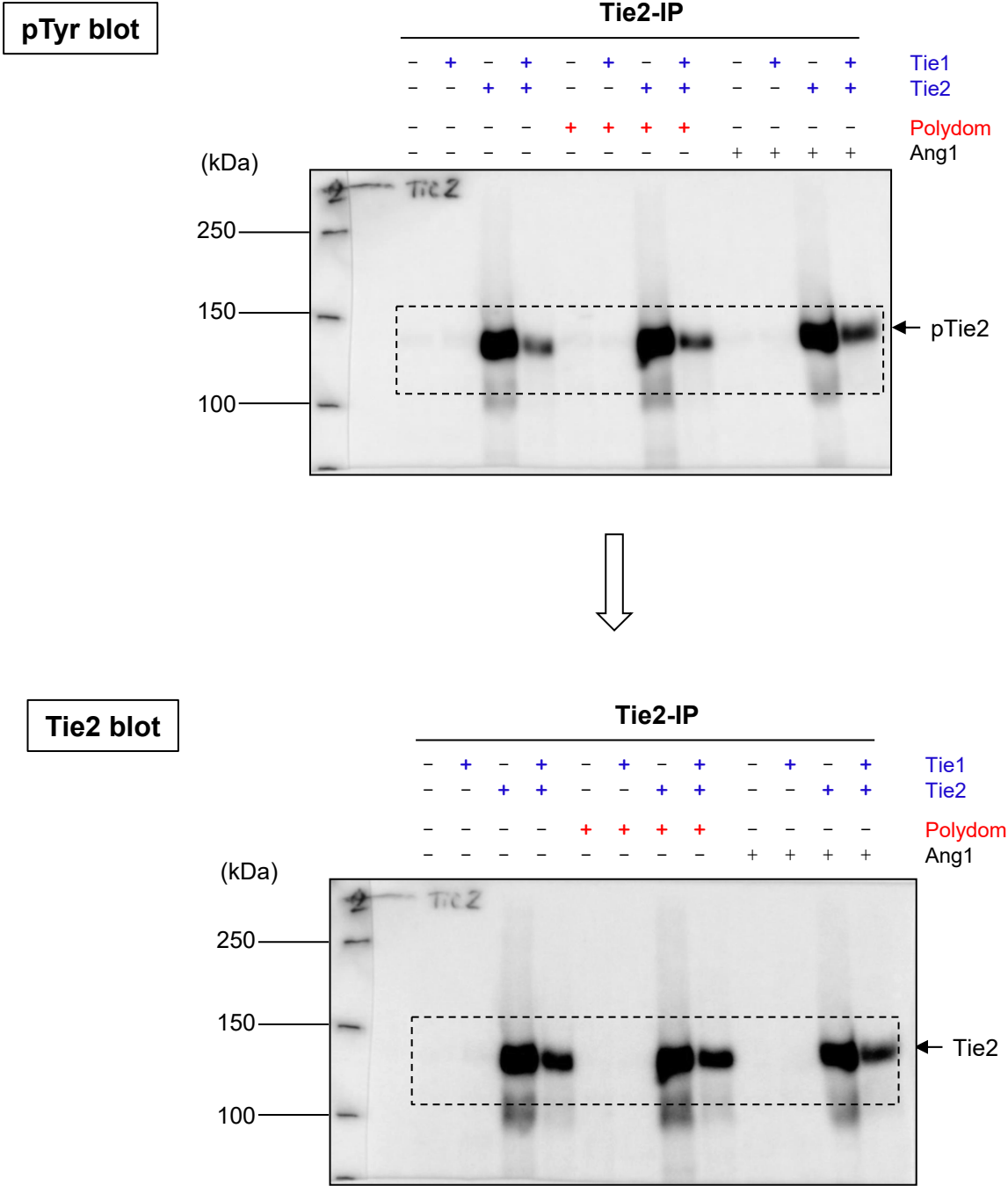

Supplement: SourceData FS3 — is the source file for Fig. S3. [file JCB_202208047_SourceDataFS3.pdf]
